# A Serological Survey of Akabane Virus Infection in Cattle in Sudan

**DOI:** 10.1155/2014/123904

**Published:** 2014-01-21

**Authors:** Amira M. Elhassan, Mohammed E. A. Mansour, Awadia A. A. Shamon, A. M. El Hussein

**Affiliations:** Veterinary Research Institute, P.O. Box 8067, Khartoum, Sudan

## Abstract

A cross-sectional survey was carried out in ten states in Sudan to determine seroprevalence and to assess risk factors associated with Akabane virus (AKAV) infection in dairy herds. Serum samples were collected from a total of 361 dairy cattle and tested for antibodies against AKAV using ELISA. The prevalence rates of AKAV antibodies in cattle varied between 69.6% in Khartoum state and 3.3% in Sennar State with an overall prevalence rate of 29.4%. The prevalence rates of AKAV antibodies were significantly associated with breed being high in crossbred (39.9%; *P* < 0.001); female sex (33%; *P* < 0.001), and animals in the age group of 2-3 years old (45.3%; *P* < 0.001). Akabane virus antibodies prevalence was also highly associated with locality (*P* < 0.001); season being high in winter season (58.1%; *P* < 0.001); and animals raised under intensive management system (37%; *P* < 0.001). Among 68 cases suffering from reproductive (abortion and infertility) problems the prevalence rate of AKAV antibodies in animals with infertility problem (76.2%; *P* < 0.03) was significantly higher than in animals with abortion (48.9%). The study revealed that AKAV infection is highly prevalent in dairy cattle in Sudan and this calls for control strategy to be implemented.

## 1. Introduction

Akabane virus (AKAV) is an arthropod-borne virus belonging to the genus *Orthobunyavirus* in the family Bunyaviridae. It causes epizootic and sporadic outbreaks of abortions, premature births, stillbirths, and congenital abnormalities characterized by arthrogryposis, hydranencephaly, or microanencephaly in cattle, sheep, and goats [1]. The virus is transmitted primarily by biting midges of *Culicoides* species [2].

AKAV is widely distributed in the tropical and temperate zones in Australia [3, 4], Southeast Asia [5], East Asia [6, 7], the Middle East [8], and Africa [9].

AKAV infection of adult cattle causes a transient viraemia without obvious clinical signs, while infection of pregnant cattle often causes fetal damage resulting in abortion, stillbirth, or various congenital abnormalities [10]. During 1972 to 1975, this disease had led to the birth of more than 42,000 abnormal calves in Japan, causing significant economic loss in cattle industry [6, 11]. Subsequently, major outbreaks resulting in congenital defects in ruminants caused by AKAV were reported from Israel, Australia, Taiwan, Korea, and Turkey [7, 10, 12–15].

In Sudan, AKAV infection was recognized based on serological evidence in sheep, goats, and cattle in various ecological zones of the country [16].

The present study aimed at investigating the current situation of AKAV infection in Sudan as a part of an ongoing research project on viral causes of reproductive problems of cattle in the country.

## 2. Materials and Methods

### 2.1. Study Area

The survey was conducted in ten states in Sudan. The investigation area extends from the latitudes 11° 78′ to 19° 61′ north and longitudes 22° 45′ to 37° 21′ east. This area is desert in the north and savannah in the south. Sera were collected from dairy cattle in White Nile, Kassala, North Kordofan, Sennar, Blue Nile, North Darfur, Red Sea, Gadarf, Khartoum, and Gazira states.

### 2.2. Study Design

This is a cross-sectional survey that included ten states of Sudan. Sample size estimation was calculated using the formula *n* = 4*PQ*/*L* ^2^ [17], where *n* is the required number of individuals to be examined; *P* is a known or estimated prevalence; *Q* = (1 − *P*); *L* is the allowable error. The number of animals estimated using this formula assuming 15% prevalence rate was 193. Since location to location variation was expected [16], the sample size was increased and all collected sera (*n* = 361) were tested (Table 1).

### 2.3. Samples

Blood samples were collected from 361 dairy cattle in 10 states of Sudan (Table 1). Animal sampled included individuals with history of abortion and infertility from two states, namely, Khartoum and Gazira states. Animals of both sexes and various age groups (less than 6 months, less than 1 year, 1-2 years, 2-3 years, and more than 3 years); breeds (crossbred and indigenous) under various management systems (extensive, semi-intensive, and intensive) and in different seasons including dry (March–June); rainy (July–October), and winter (November–February) were bled. Sera were extracted by centrifugation at 1,500 rpm for 10 minutes and kept at −20°C until they were tested for presence of antibodies against AKAV using ELISA.

A total of 68 out of the 361 sera samples were collected from animals suffering from reproductive problems (infertility and abortion).

### 2.4. ELISA for Detection of AKAV Antibodies

Commercial competitive ELISA antibodies kit (ID VET innovative diagnostics, France) was used to detect antibodies (anti-g1) against AKAV according to manufacturer's instructions.

### 2.5. Statistical Analysis

The serological results and other information gathered during this investigation such as locality, sex, season, management system, signs of reproductive problem, and breed (indigenous and crossbred) of the sampled animals were edited and analyzed statistically using statistical package (SPSS version 16). To identify the association of the risk factors with the specific seroprevalence, the Chi-square (*χ* ^2^ test) was used. The statistical significance level used was *P* ≤ 0.05.

## 3. Results 

The overall prevalence rate of AKAV antibodies found in dairy cattle samples was 29.4% (106/361) (Table 1). The prevalence rate varied from highest (69.6%) (64/92) in Khartoum State to lowest (3.3%) (1/30) in Sennar State. The prevalence rate was significantly variable among states (*P* < 0.001).

Likewise the prevalence of AKAV antibodies was significantly (*P* < 0.001) higher in crossbred animals (39.9%) than in indigenous breed animals (8.9%) and in females (33%) than in males (14.3%; *P* < 0.001). The prevalence rates were significantly higher in intensively (37%) managed animals (*P* < 0.001) (Table 2). The seroprevalence was significantly high among 2-3 years old (45.3; *P* < 0.001) and in the winter season (58.1%; *P* < 0.001).

The prevalence rates were also high in cases of infertility (76.2%) and abortion (48.9%) when compared to apparently healthy animals (22.9%) and were significantly associated with cases of infertility (*P* < 0.03) (Table 3).

## 4. Discussion

An earlier investigation by Mohamed et al. [16] conducted to assess prevalence of AKAV antibodies in domestic ruminants in different ecological zones of Sudan indicated prevalence rates of up to 27% in sheep, 36% in goats, and 47% in cattle. The prevalence rates in cattle ranged between 10.6% and 47.8% in different areas [16]. In the present study, prevalence rates of AKAV antibodies in dairy cattle varied greatly ranging from 3.3% in Sennar State to 69.6% in Khartoum State with an overall prevalence rate of 29.4%.

This higher seroprevalence in Khartoum State may be due to the fact that in this state 59 cattle sera out of 92 serum samples investigated were suffering from abortion (39 cases) and infertility (20 cases) (Table 3). Subsequently, the seropositivity of AKAV was high among cattle in this state. This result confirmed records of Jun et al. [18] who discussed the associations between this virus and abortion suggesting that this virus may significantly increase abortion risk among cattle and sheep.

Mohamed et al. [16] stated that transmission of AKAV in Sudan may not be an annual event with more sporadic occurrences, a fact that may lead to variations in prevalence rates from year to year and from one area to another. Thus, while these authors recorded a prevalence rate of 10.6% for Sennar State, the prevalence recorded in the present study was 3.3% for the same area. However, both studies indicated wide geographic distribution of AKAV seropositivity in cattle in Sudan indicating that the vector(s) of AKA virus are widespread; unfortunately information regarding the vector(s) in the country is lacking. It is interesting, however, that, inspite of the observed high prevalence and wide distribution of AKAV infection in Sudan, no evidence of outbreaks of congenital malformations, as has been reported in other parts of the world, has been recorded in dairy cattle in Sudan. This may be related to strain(s) of the virus circulating in Sudan.

The present study also revealed that prevalence rates were highly associated with locality, breed, sex, season, age of animals, and management system. The prevalence rate was significantly higher in Khartoum State (69.6%; *P* < 0.001) than in other States. Animals sampled in Khartoum State were from intensively managed crossbred dairy cattle that reported reproductive problems based on a questionnaire survey administered prior to collection of samples in Khartoum and in Gazira States. The prevalence rates were also significantly higher in crossbred animal (39.9%; *P* < 0.001) than in indigenous ones (8.9%) and in females (33%; *P* < 0.001) than in males which may reflect higher degree of attractiveness of these animals to insect vectors. The prevalence rates were also higher in intensive and semi-intensive management systems (37.0% and 36.1%, resp.) than in extensive systems. The previous two systems are generally concentrated around urban areas, and crossbred animals usually constitute the majority of animals in these systems while extensive systems are usually practiced in the vast grass lands of eastern and western Sudan and usually involve indigenous animals. In addition vector arthropods may not be as numerous in the extensive system as in the intensive and semi-intensive system where microenvironmental conditions suitable for vector breeding and survival may be available year-round. This study also revealed that the season represents a risk factor of AKAV transmission. Seroprevalence rates were much higher in winter season (58.1%) than in dry (25.6%) or rainy (25.3%) season. Winter season in much of the Sudan is usually very mild with an average temperature that is usually above 30°C. These conditions might be conducive for vector breeding and survival.

When examining prevalence rates of AKAV antibodies in individuals with reproductive problems, the results revealed that these rates were significantly higher in cases of infertility (76.2%; *P* < 0.03) than in abortion cases (48.9%) and were in both cases higher than in apparently healthy (22.9%) animals. However, the high prevalence rates observed in both infertility and abortion cases may indicate the importance of AKAV infection in the bovine reproductive syndrome in Sudan warranting further investigations.

## 5. Conclusion

The present study clearly indicated the widespread prevalence of AKAV infection in dairy cattle in Sudan. Absence of clinically recognized symptoms of AKAV infections with noticeable outbreaks of abortion and foetal malformations may lead to underestimation of the importance of the disease in the Sudan. This situation may, however, change and the disease can pose considerable threat with the increasing trend toward intensive raising of exotic breed dairy cattle to satisfy the rising local demand for milk and other dairy products.

Hence, further countrywide epizootiological studies of AKAV infections in cattle, sheep, and goats are called for to provide information required for formulating well-designed control programs.

## Figures and Tables

**Table 1:** Prevalence of AKAV antibodies using cELISA among cattle in different States in Sudan.

Location	No. examined	AKA positive (%)
Khartoum	92	64 (69.6%)*
White Nile	30	3 (10%)
Kassala	10	1 (10%)
North Kordofan	30	2 (6.6%)
Sennar	30	1 (3.3%)
Blue Nile	31	3 (9.7%)
North Darfur	30	6 (20%)
Red Sea	28	9 (45%)
Gadarif	26	1 (3.8%)
Gazira	54	16 (29.6%)

Total	361	106 (29.4%)

*P value < 0.001.

**Table 2:** Influence of some risk factor on seroprevalence of AKAV in cattle from different States in Sudan.

Variable	Group	No. of samples	Percentage positive
Breed	Indigenous	123	11 (8.9%)
	Cross	238	95 (39.9%)*

Sex	Female	291	96 (33%)*
	Male	70	10 (14.3%)

Age	Less than 6 months	43	4 (9.3%)
	Less than 1 year	54	5 (9.3%)
	1-2 years	51	15 (29.4%)
	2-3 years	75	34 (45.3%)*
	More than 3 years	138	48 (34.8%)

Management	Extensive	100	11 (11%)
	Semi-intensive	180	65 (36.1%)
	Intensive	81	30 (37.0%)*

Season	Dry (March–June)	227	58 (25.6%)
	Rainy (July–October)	91	23 (25.3%)
	Winter (November–February)	43	25 (58.1%)*

*P value < 0.001.

**Table 3:** Association between results of AKAV and signs of reproductive problem in cattle from Khartoum and Gazira States.

State	Abortion		Total	Infertility		Total
	Positive (%)	Negative (%)		Positive (%)	Negative (%)	
Khartoum State	24 (61.5)	15 (28.5)	39	15 (75)	5 (25)	20
Gazira State	0 (0)	8 (100)	8	1 (100)	0 (0)	1

Total			47			21
